# Current Status, Challenges, and Opportunities Associated With Implementing Clinical Data Interchange Standards Consortium Standards in Japanese Academic Medical Centers: Cross-Sectional Survey

**DOI:** 10.2196/83774

**Published:** 2026-03-06

**Authors:** Kaori Nagai, Noriko Ito, Yuya Ikeda, Toshiki I Saito

**Affiliations:** 1 Clinical Research Center, NHO Nagoya Medical Center, 4-1-1 Sannomaru, Naka-ku, Nagoya, Aichi, 460-0001, Japan, 81 52-951-1111; 2 Clinical Research Support Office, National Cancer Center Hospital East, Kashiwa, Chiba, Japan; 3 Clinical Research Center, NHO Headquarters, Meguro-ku, Tokyo, Japan

**Keywords:** Clinical Data Interchange Standards Consortium, CDISC standards, academic research organization, ARO, Clinical Data Acquisition Standards Harmonization, CDASH, Study Data Tabulation Model, SDTM, Analysis Data Model, ADaM

## Abstract

**Background:**

The implementation of Clinical Data Interchange Standards Consortium (CDISC) standards is essential for accelerating clinical research and is mandated for new drug applications in Japan. However, the current status of their implementation and associated challenges in Japanese academic medical centers has not been comprehensively investigated.

**Objectives:**

This study aimed to comprehensively clarify the current status, challenges, and opportunities associated with implementing CDISC standards in Japanese academic medical centers.

**Methods:**

We conducted a questionnaire survey targeting 91 academic medical centers (83 university hospitals, 7 national centers for advanced and specialized medical care, and 1 national hospital organization) from June to August 2024. The survey assessed experience with contracted CDISC data creation, implementation status, barriers, and available resources. We performed Mann-Whitney *U* tests to assess the association between the number of data managers or biostatisticians and CDISC implementation status.

**Results:**

Responses were received from 84 institutions (response rate: 92%). While 29% (26/91) had experience with contracted CDISC standards data creation, 22% (20/91) had implemented CDISC standards. The most frequently cited barrier to CDISC standards implementation was “significant human and financial effort and resource shortages” (62%, 56/91). A significant “supply and demand” gap was identified: while 66% (60/91) of institutions expected support (eg, materials, workshops) from other organizations, only 5% (5/91) were capable of providing such resources. Furthermore, 66% (60/91) of institutions had not yet decided on a future policy regarding CDISC compliance. Notably, institutions that had implemented CDISC standards had significantly higher numbers of data managers (*P*<.001) and biostatisticians (*P*<.001) compared to those that had not.

**Conclusions:**

The implementation of CDISC standards in Japanese academia is limited, primarily due to resource shortages and a lack of specialized personnel. However, the discrepancy between the high demand for support and limited supply highlights a significant opportunity. Experienced academic research organizations, in cooperation with pharmaceutical companies and contract research organizations, can drive nationwide adoption by taking a strategic leadership role in providing educational resources and training, thereby addressing the critical shortage of specialized personnel and know-how.

## Introduction

The Clinical Data Interchange Standards Consortium (CDISC) is a nonprofit organization dedicated to developing global electronic standards for clinical trial data. The “CDISC standards” refer to the set of standards developed by this consortium. Implementing these standards entails a series of technical activities throughout the clinical data lifecycle. Specifically, it involves designing case report forms based on Clinical Data Acquisition Standards Harmonization (CDASH), mapping collected raw data to Study Data Tabulation Model (SDTM) domains, and creating analysis-ready datasets according to Analysis Data Model (ADaM). These processes require writing programming scripts for data transformation, generating metadata files such as define.xml, and rigorously validating the datasets and their conformance to the standards. In Japan, the Pharmaceuticals and Medical Devices Agency (PMDA) has been accepting electronic clinical trial data submissions compliant with CDISC standards for new drug marketing approval applications since October 2016 [1]. Furthermore, the Japan Agency for Medical Research and Development has expressed the view that compliance with CDISC standards from the planning and execution stages will become essential in academia-led clinical research and trials in the future [2].

Additionally, global data sharing platforms promote the sharing and reuse of clinical trial data [3-9], and several of these advance data standardization in collaboration with the CDISC [3-6,8].

The sharing of clinical trial data is expected to promote advancements in medicine and science [10,11]. Moreover, since July 1, 2018, the International Committee of Medical Journal Editors has mandated the inclusion of a data-sharing statement in manuscripts reporting the results of clinical trials submitted to International Committee of Medical Journal Editors member journals [12].

In 2017 in Japan, the University Hospital Medical Information Network Center and Japan Agency for Medical Research and Development cohosted the “CDISC Public Symposium” [2]. Furthermore, in 2018, University Hospital Medical Information Network, PMDA, and CDISC cohosted the “Workshop on CDISC Utilization in Academia” [13]. These activities aim to promote the dissemination of CDISC standards in Japanese academia.

The implementation of CDISC standards is expected to accelerate clinical research [14]. In particular, researchers and related institutions are beginning to recognize the importance of planning research and preparing case report forms based on the premise of electronic submissions compliant with CDISC standards throughout the processes of data collection, data management, and statistical analysis. These initiatives are expected to help reduce costs and workloads while maintaining data quality [15].

A previous study investigated the current status and challenges associated with the implementation of CDISC standards in Japanese academic medical centers [16]. However, in that study, the definition of “academia” was ambiguous.

Therefore, the present study aimed to comprehensively clarify the current status and challenges associated with implementing CDISC standards in Japanese academic medical centers.

## Methods

### Ethical Considerations

This study was a questionnaire survey targeting administrative personnel at academic medical centers regarding the operational status of CDISC standards. The study did not involve interventions on human participants or the collection of sensitive personal health information from patients. Therefore, an ethics review board assessment was not sought, in accordance with the ethical guidelines for medical and health research involving human subjects in Japan.

The purpose of the study was clearly explained in the invitation to the online questionnaire, and the completion and submission of the questionnaire were regarded as implied consent to participate. Participation was voluntary, and respondents could withdraw at any time by not submitting their responses.

To protect the privacy and confidentiality of the respondents, the collected data were stored securely and accessed only by the research team. The data were aggregated for analysis, and no personally identifiable information was reported. No compensation was provided to the participants for this study. This manuscript and the supplementary materials do not contain any images or data that could identify individual participants.

### Participants and Survey Administration

In this study, “academia” is defined as institutions in Japan legally mandated to conduct research as part of their official duties. Based on this definition, the following 91 academic medical centers (hereinafter referred to as “institutions”) were selected as survey targets: 83 university hospitals (43 national university hospitals, 8 public university hospitals, 31 private university hospitals, and 1 ministry-affiliated university hospital), 7 national centers for advanced and specialized medical care, and 1 national hospital organization. We contacted these institutions by email, requested their cooperation in the questionnaire survey, and sent them the questionnaire.

The survey period was from June 12 to August 16, 2024. The questionnaire survey was conducted online using Google Forms. As a general rule, 1 response per institution was accepted. No duplicate responses were received from any single institution; therefore, all submitted responses were included in the final analysis.

The survey was primarily distributed to the directors of clinical research centers or heads of data centers at each institution. In cases where direct contact information for these roles was unavailable, we identified the appropriate respondents through personal professional networks. For institutions where no direct contact could be established, the request was sent to the general administrative office of the Clinical Research Support Center, with instructions to forward the questionnaire to the staff member most knowledgeable about CDISC standards.

### Questionnaire Items

The main questionnaire items were as follows:

・Experience with contracted CDISC standards data creation

・Implementation status of CDISC standards

・Barriers to CDISC standards implementation

・Future policies regarding CDISC standards-compliant data creation

・Availability of CDISC standards-related documents and training

・Ability to provide CDISC standards-related documents and training

・Expectations for pharmaceutical companies, contract research organizations (CROs), and other academic research organizations (AROs)

・Ability to provide to pharmaceutical companies, CROs, and other AROs

・Number of data managers and biostatisticians

In this study, “implementation” was defined as CDISC data creation conducted internally, jointly with external partners, or externally with acceptance reviews performed by internal CDISC experts. “Experience with contracted CDISC standards data creation” was defined more broadly to encompass these implementation categories as well as external data creation performed without such internal expert-led acceptance procedures.

Further details regarding the questionnaire items are presented in Multimedia Appendix 1.

### Statistical Analysis

Descriptive statistics were calculated using the total number of targeted institutions (n=91) as the denominator. This approach was adopted to establish a consistent baseline for future longitudinal surveys targeting the same population. Furthermore, using the total population as the denominator prevents the overestimation of implementation rates caused by selection bias—where institutions with greater interest in CDISC standards are more likely to respond—and ensures the results accurately reflect the status across all defined Japanese institutions. Categorical variables are presented as numbers and percentages. To assess the association of the number of data managers and biostatisticians with the CDISC implementation status, we performed the Mann-Whitney *U* test. In this analysis, the number of staff was treated as a continuous variable. Although staffing levels were categorized (0‐3, 4‐10, and ≥11) for visualization purposes, the statistical hypothesis testing was based on the continuous data. To examine potential confounders, we also analyzed the association between institution type (public/national vs private) and CDISC implementation using Fisher exact test. A *P* value <.05 was considered statistically significant. All statistical analyses were performed using R statistical software (version 4.4.1; R Foundation for Statistical Computing).

## Results

### Response Rate and Participant Characteristics

In this study, 91 institutions were targeted, among which responses were received from 84 (response rate: 92%). Of these, 7 (8%) institutions did not respond. The breakdown of the responding institutions was as follows: 42 national university hospitals, 27 private university hospitals, 7 public university hospitals, 7 national centers for advanced and specialized medical care, and 1 national hospital organization. No responses were received from ministry-affiliated university hospitals. The breakdown of respondents by job title is shown in Multimedia Appendix 2.

The percentages in these results were calculated with the total number of targeted institutions (N=91) as the denominator and rounded to the nearest integer, unless otherwise specified.

### Experience With Contracted CDISC Standards Data Creation

**Figure 1.**
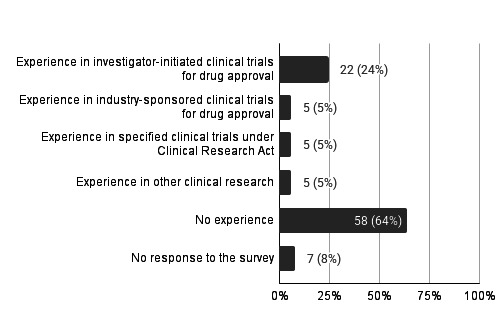
Experience with contracted Clinical Data Interchange Standards Consortium standards data creation (multiple responses allowed) (N=91).

Experience with contracted CDISC standards data creation refers to the track record of accepting projects for CDISC data creation. This includes cases where the actual data creation was outsourced to a third party. Regarding experience with contracted data creation using CDISC standards, 26 (29%) institutions reported having such experience, while 58 (64%) did not; 7 (8%) institutions did not respond to this question. Among the types of trials for which institutions had experience (multiple answers allowed), investigator-initiated clinical trials for drug approval were the most common, at 22 (24%), followed by industry-sponsored clinical trials for drug approval (n=5, 5%), specified clinical trials under Japan’s Clinical Research Act [17] (n=5, 5%), and other clinical research (n=5, 5%) (Figure 1).

These results indicate that slightly less than 30% (26/91) of the institutions had experience with contracted data creation using CDISC standards. Notably, this experience extends beyond trials for drug approval to include specified clinical trials and other clinical research.

### Implementation Status of CDISC Standards

Implementation of CDISC standards refers to creating CDISC data using internal resources or, in the case of outsourcing, conducting acceptance work by CDISC experts. Regarding the implementation status of CDISC standards, 20 institutions (22%) reported having implemented them, whereas 64 institutions (70%) reported not having implemented them; seven institutions (8%) did not respond.

**Figure 2.**
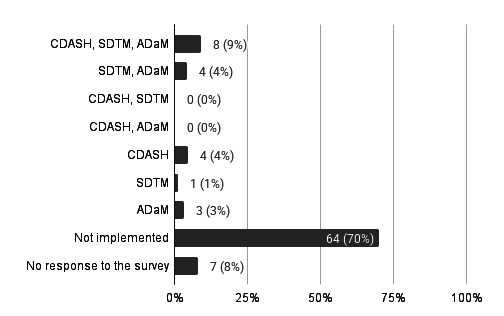
Implementation status of the Clinical Data Interchange Standards Consortium standards (N=91). ADaM: Analysis Data Model; CDASH: Clinical Data Acquisition Standards Harmonization; SDTM: Study Data Tabulation Model.

Among the 20 institutions that had implemented CDISC standards, the most common combination of standards (multiple answers allowed) was the implementation of all three—CDASH, the SDTM, and the ADaM—reported by eight institutions (9% of the 91 institutions). This was followed by institutions implementing SDTM and ADaM (n=4, 4%), CDASH only (n=4, 4%), ADaM only (n=3, 3%), and SDTM only (n=1, 1%) (Figure 2).

Regarding individual standards, ADaM was implemented by 15 (16%) institutions, SDTM by 13 (14%), and CDASH by 12 (13%).

These results indicate that while only 22% (20/91) of all institutions had implemented CDISC standards, many of these implementing institutions use multiple standards (CDASH, SDTM, and ADaM) in combination.

### Barriers to CDISC Standards Implementation

**Figure 3.**
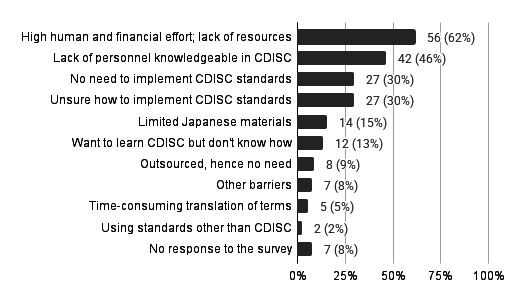
Barriers to CDISC standards implementation (multiple responses allowed). Note: Percentages were calculated based on the total number of survey targets (N=91). “No response to the survey” indicates institutions that did not answer this survey entirely. CDISC: Clinical Data Interchange Standards Consortium.

Barriers with CDISC implementation refer to the obstacles or challenges that institutions face when attempting to adopt or maintain CDISC standards. Institutions cited the following items as barriers to CDISC standards implementation (multiple answers allowed). The most frequently cited barrier was “high human and financial effort and lack of resources” (n=56, 62%), followed by “lack of personnel knowledgeable in CDISC” (n=42, 46%), “no need to implement CDISC standards” (n=27, 30%), and “unsure how to implement CDISC standards” (n=27, 30%) (Figure 3).

These results indicate that the main barriers to CDISC standards implementation are the significant human and financial effort required, resource shortages, and a lack of personnel knowledgeable about CDISC.

### Future Policies Regarding CDISC Standards-Compliant Data Creation

Regarding future policies for CDISC standards-compliant data creation (single answer), the most common response, reported by 60 (66%) institutions, was “no decision made,” followed by “will not undertake” (n=7, 8%), “will undertake, and external experts will perform the work with internal involvement” (n=6, 7%), “will undertake, and both internal and external personnel will perform the work” (n=5, 5%), “will undertake, but only external experts will perform the work without internal involvement” (n=5, 5%), and “will undertake, and the work will be performed internally” (n=1, 1%) (Table 1). These results indicated that 66% (60/91) of the institutions had not yet decided on their future policy; only 19% (17/91) had a clear policy for undertaking such work.

**Table 1.** Future policies regarding CDISC^a^ standards-compliant data creation (N=91).^b^

Future policy regarding CDISC compliance	All institutions (N=91), n (%)	Currently implemented (n=20), n (%)	Currently not implemented (n=64), n (%)
Internal execution	1 (1)	1 (5)	0 (0)
Joint internal and external execution	5 (5)	3 (15)	2 (3)
External execution with internal involvement	6 (7)	5 (25)	1 (2)
External execution without internal involvement	5 (5)	1 (5)	4 (6)
Will not undertake	7 (8)	2 (10)	5 (8)
No decision made	60 (66)	8 (40)	52 (81)
No response to the survey	7 (8)	—^c^	—

a CDISC: Clinical Data Interchange Standards Consortium.

b Note: All values are n (%); percentages are calculated based on the total number of survey targets (N=91). “Implemented” and “not implemented” columns represent the current status at the time of the survey, where “implementation” is defined as internal CDISC data creation or expert-led acceptance work for outsourced tasks. “No response to the survey” refers to institutions that did not return the questionnaire.

c Not available.

The association between current implementation status and future policies is detailed in Table 1. Among the 20 institutions that had already implemented the standards, fewer than half (9 institutions, 45%) intended to continue “implementation” (defined as work involving internal resources or internal expert-led acceptance). A similar lack of interest was observed among the 64 nonimplementing institutions, with only 3 (5%) planning for future implementation.

### CDISC Standards Implementation Resources: Availability and Ability to Provide

Among all, 15 (16%) institutions reported having documents or training related to implementing CDISC standards. A breakdown of these resources (multiple answers allowed) indicated that “beginner-level training” was the most common, reported by 12 (13%) institutions, followed by “Japanese-language educational materials” (n=9, 10%), “documents on implementation know-how” (n=8, 9%), and “tools supporting CDISC” (n=8, 9%) (Table 2).

**Table 2.** Availability of and ability to provide CDISC^a^ standards-related documents and training (multiple responses allowed) (N=91).^b^

Item	Availability, n (%)	Ability to provide, n (%)
Japanese-language educational materials on CDISC standards	9 (10)	4 (4)
Documents on implementation know-how for CDISC standards	8 (9)	4 (4)
Tools supporting CDISC standards implementation	8 (9)	4 (4)
Beginner-level training on CDISC standards	12 (13)	5 (5)
No response to the survey	7 (8)	7 (8)

a CDISC: Clinical Data Interchange Standards Consortium.

b Note: Percentages were calculated based on the total number of survey targets (N=91). "No response to the survey" indicates institutions that did not answer this survey entirely.

Next, regarding the question of whether these documents and training could be provided to other institutions, 5 (5%) institutions responded that they “could provide” them. A breakdown of providable resources is shown in Table 2.

These results indicate that while 16% (15/91) of institutions had CDISC standards implementation resources available, only 5% (5/91) were able to provide these resources to other institutions.

### Support Expected From and Providable to Pharmaceutical Companies, CROs, and Other AROs

Regarding expectations for pharmaceutical companies, CROs, and other AROs (multiple answers allowed), 60 (66%) institutions responded that they had “some expectations.” Among these 60 institutions, the most desired item was “materials and information on CDISC” (n=53, 58%), followed by “training sessions (seminars, workshops, etc) on CDISC” (n=45, 49%) and “on-the-job training related to CDISC” (n=33, 36%) (Table 3).

**Table 3.** Expectations for and ability to provide support to pharmaceutical companies, contract research organizations, and other academic research organizations (multiple responses allowed) (N=91).^a^

Item	Expectations, n (%)	Ability to provide, n (%)
Training sessions (seminars, workshops, etc) on CDISC^b^	45 (49)	2 (2)
On-the-job training related to CDISC	33 (36)	2 (2)
Materials and information on CDISC	53 (58)	4 (4)
No response to the survey	7 (8)	7 (8)

a Note: Percentages were calculated based on the total number of survey targets (N=91). "No response to the survey" indicates institutions that did not answer this survey entirely.

b CDISC: Clinical Data Interchange Standards Consortium.

Next, regarding support that could be provided to pharmaceutical companies, CROs, and other AROs (multiple answers allowed), 5 (5%) institutions responded that they “could provide” some support. A breakdown is shown in Table 3.

These findings revealed that 66% (60/91) of the institutions expected CDISC-related material provision, information sharing, and workshop hosting from pharmaceutical companies, CROs, and other AROs. On the other hand, only 5% (5/91) of the institutions were able to provide such support, indicating a limited capacity.

### Number of Data Managers and Biostatisticians

**Figure 4.**
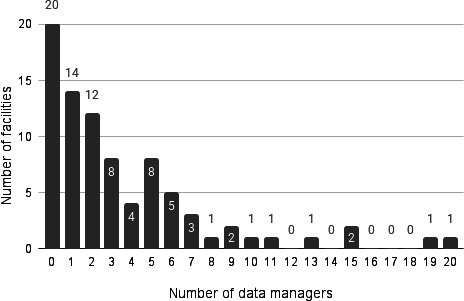
Number of data managers at responding facilities (n=84). Note: Analysis excluded the 7 institutions that did not respond to the survey.

**Figure 5.**
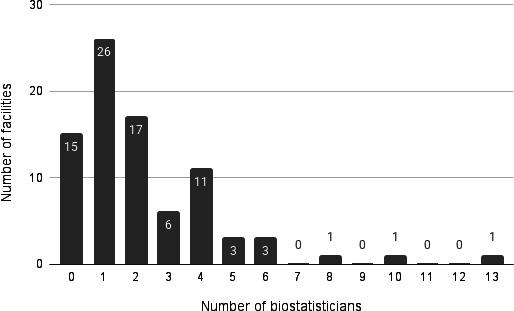
Number of biostatisticians at responding facilities (n=84). Note: Analysis excluded the 7 institutions that did not respond to the survey.

Among the 84 responding institutions, the median number of data managers was 2 (mode: 0; range: 0‐20) (Figure 4). Similarly, the median number of biostatisticians was 2 (mode: 1, range: 0‐13) (Figure 5).

### Association Between Staffing Levels and CDISC Standards Implementation Status

**Figure 6.**
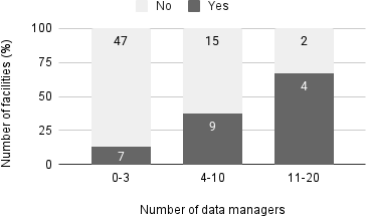
Number of data managers and status of Clinical Data Interchange Standards Consortium standards implementation (n=84). Note: Analysis excluded the 7 institutions that did not respond to the survey. Data were categorized for visualization purposes, while the Mann-Whitney *U* test was performed on continuous variables (*P*<.001).

**Figure 7.**
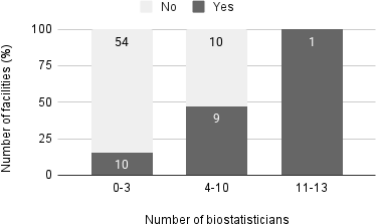
Number of biostatisticians and status of Clinical Data Interchange Standards Consortium standards implementation (n=84). Note: Analysis excluded the 7 institutions that did not respond to the survey. Data were categorized for visualization purposes, while the Mann-Whitney *U* test was performed on continuous variables (*P*<.001).

An analysis of the relationship between staffing levels and CDISC implementation showed that institutions with more data managers tended to be more advanced in their implementation (median 5, IQR 2‐9.25 vs median 2, IQR 0‐4; *P*<.001) (Figure 6). A similar trend was observed for biostatisticians (median 3.5, IQR 1.75‐5 vs median 1, IQR 1-2; *P*<.001) (Figure 7).

These findings suggest that institutions with more data managers and biostatisticians tend to be further along in implementing CDISC standards.

In addition to staffing levels, we evaluated the association between institution type and CDISC implementation. Implementation rates were 28.1% (16/57) in Public/National institutions and 14.8% (4/27) in private institutions; however, no significant difference was observed (Fisher exact test, *P*=.27).

## Discussion

### Current Status and Significance of CDISC Standards Implementation

In a questionnaire survey conducted in 2020 to clarify the current status of and challenges associated with CDISC standards implementation in Japanese academic medical centers [16], the definition of “academia” was ambiguous, which posed challenges in interpreting the results and tracking changes over time. Therefore, in the present study, “academia” was more strictly defined to ensure the accuracy of longitudinal comparisons. By selecting institutions based on this clear definition, the present study could assess the current status and challenges associated with implementing CDISC standards more accurately, thereby providing foundational data to promote future adoption.

In a questionnaire survey conducted in 2020 to clarify the current status of and challenges associated with CDISC standards implementation in Japanese academic medical centers [16], the definition of “academia” was ambiguous, which posed challenges in interpreting the results and tracking changes over time. Therefore, in the present study, “academia” was more strictly defined to ensure the accuracy of longitudinal comparisons. By selecting institutions based on this clear definition, the present study could assess the current status and challenges associated with implementing CDISC standards more accurately, thereby providing foundational data to promote future adoption.

The findings revealed that only 29% (26/91) of institutions had experience with contracted CDISC standards data creation. Furthermore, institutions with experience in contracted data creation most frequently undertook investigator-initiated clinical trials for drug approval. This finding suggests that the application of CDISC standards in Japan, particularly that centered around investigator-initiated clinical trials for drug approval, is progressing. On the other hand, the present findings also confirmed that institutions had some experience in contracted CDISC standards data creation for specified clinical trials under the Clinical Research Act and other clinical research not intended for marketing approval applications. These findings suggest that CDISC standards are being used not only for regulatory submissions but also for standardizing and improving the efficiency of overall clinical research operations. In addition, it has been reported internationally that CDISC standards are also being used for Real-World Data [18]. We believe that clarifying the significance of standardization and efficiency and sharing practical case studies could lead to the promotion of CDISC standards implementation.

Regarding the implementation status of CDISC standards, the present study found that only 22% (20/91) of institutions had implemented them, indicating limited adoption. Notably, this study confirmed that while a high proportion of implementing institutions adopt multiple CDISC standards in combination, a certain number of institutions implement only individual standards. These results suggest that varying needs and resource situations at each institution may influence the form of CDISC standards implementation.

### Barriers to CDISC Standards Implementation

Regarding implementation barriers, this study confirmed that “human and financial effort and resource shortages” remains the greatest challenge, followed by “a lack of personnel knowledgeable about CDISC.” This finding suggests that securing human and financial resources and developing CDISC specialist personnel within institutions are crucial for promoting CDISC standards implementation. The findings also revealed that only 16% (15/91) of institutions possess documents or training related to CDISC standards, and furthermore, only 5% (5/91) of institutions can provide these resources to other institutions, which suggests that the expansion of CDISC-related educational resources within the country remains a challenge. We hypothesize that this discrepancy may stem from proprietary restrictions, copyright issues, or the lack of generalized templates that can be easily shared across institutions. In AROs in other countries as well, there is a demand not only for formal training by experts but also for open-source resources and “bite-sized” educational materials that are easily accessible online, such as the W3Schools model, as suggested by Jentoft et al [19]. Therefore, developing similar practical and accessible educational resources may serve as an important strategy for addressing the shortage of training materials in Japan.

Regarding barriers to implementation, 27 (30%) institutions cited “no need to implement CDISC standards.” Within this group, some respondents cited “outsourced, hence no need.” Furthermore, our detailed analysis revealed that the majority of these 27 (81%, 22/27) institutions selected “will not undertake” or “no decision made” regarding their future policies on CDISC standards-compliant data creation. The primary reason for these responses is likely that the PMDA requires CDISC compliance exclusively for new drug marketing approval applications, whereas many institutions primarily conduct research under the Clinical Research Act that falls outside this mandate. We included these institutions in our analysis to accurately reflect the current status of adoption, as excluding them would overlook the reality that a significant segment of academia operates outside the regulatory scope.

Regarding the respondents who cited outsourcing, it is important to distinguish this group from institutions that outsource but are considered to have “implemented” CDISC standards. As defined in our survey, “implementation” via outsourcing requires the institution to perform acceptance work using internal CDISC experts. In contrast, we interpret that respondents viewing outsourcing as a reason for nonimplementation may rely entirely on vendors without maintaining internal standards or oversight capabilities. However, as acknowledged in the Limitations section, the lack of explicit definitions in the survey may have led to variations in respondent interpretation regarding this distinction.

### Future Policies and Opportunities for Strategic Support

Furthermore, the finding that a significant majority of institutions (66%, 60/91; Table 1) have made “no decision” regarding future CDISC policy suggests a deeper issue than mere resource constraints. Notably, this trend extends even to institutions that have already implemented CDISC standards. Half of these institutions (11/20) are uncertain about, or have decided against, continuing the implementation of CDISC-compliant data in the future, suggesting serious challenges in the sustainability of CDISC operations. This strategic inertia may indicate a systemic barrier, potentially stemming from the lack of regulatory incentives or clear mandates for the application of CDISC standards in clinical research not aimed at marketing approval. We considered that addressing this structural uncertainty is essential to enable institutions to commit to definitive standardization strategies.

While experienced AROs can play a pivotal role in driving adoption, the survey results indicate that 66% (60/91) of institutions specifically expect support from “pharmaceutical companies, CROs, and other AROs,” with a primary desire for “materials and information.” This highlights that academic institutions often cannot address the implementation gap on their own. Therefore, future strategies should not rely exclusively on peer-to-peer academic support but should also leverage industry resources. Active involvement and support from pharmaceutical companies and CROs were considered essential to provide the resources needed to bridge the gap between supply and demand.

### Association Between Staffing Levels and Implementation

The present findings revealed significant variation among institutions in terms of the number of data managers and biostatisticians on staff. However, institutions with more of these specialists tended to be further along in regard to implementing CDISC standards. This result suggests that securing personnel with specialized knowledge is important for the effective implementation of CDISC standards, and as such, data managers and biostatisticians play a central role in the implementation process.

### Limitations

A major limitation of this study is that adjustments for institutional resources such as hospital bed count or budget were not performed; these factors may be stronger determinants of CDISC adoption than staffing levels alone. The observed association between dedicated staffing and implementation may, therefore, be a reflection of overall institutional size or resource availability. Furthermore, the primary outcome variable, CDISC implementation status, was based solely on self-reported metrics from respondents and was not verified by external audit or inspection of the actual data standards used. This introduces the potential for response bias, such as the overestimation of institutional compliance. Finally, we acknowledge the nonresponse from specific institutional types; specifically, no responses were received from ministry-affiliated university hospitals. However, as this institutional category comprised only 1.1% (1/91) of the total institutions surveyed, the impact on the overall representativeness of Japanese academic medical centers is likely minimal. Furthermore, this study relies on self-reported data, which may be subject to respondent bias or misunderstanding of technical terms. For instance, a small number of sites reported implementing “ADaM only” without SDTM. Given that ADaM datasets are typically derived from SDTM, this discrepancy suggests a potential lack of accurate understanding of the standards among some respondents. Therefore, the results regarding technical proficiency should be interpreted with caution as they represent the respondents’ subjective assessments rather than objectively verified capabilities. Furthermore, the questionnaire option labeled “outsourced, hence no need” was presented without explicit definitions distinguishing between outsourcing as a form of implementation and outsourcing as a reason for nonimplementation. Consequently, respondents may have selected this option based on their own interpretation. This lack of clarity may have led to misclassification, where institutions that had successfully implemented the system via outsourcing (with internal acceptance work) categorized themselves as having “no need.”

### Recommendations and Future Work

Therefore, developing these specialized human resources is likely essential for promoting their adoption. Expanding training opportunities and improving access to information regarding CDISC standards are key to promoting their wider adoption in Japanese academic medical centers.

### Conclusions

The findings of the present study indicate that the implementation of CDISC standards in Japanese academic medical centers remains limited. The primary barrier identified was a lack of human and financial resources. On the other hand, many institutions are interested in implementing CDISC standards and seeking support such as workshops and the provision of materials. To bridge the gap between this high demand and the limited supply, future strategies should involve not only peer-to-peer academic support but also active resource sharing and cooperation from pharmaceutical companies and CROs. Notably, instances were observed where CDISC standards were being used even in clinical research not directly aimed at marketing approval applications, which is interpreted as a preliminary indication of the potential recognition within the research community of the operational efficiencies gained through standardization. Furthermore, the present findings suggest that the number of data managers and biostatisticians on staff is strongly associated with the implementation status of CDISC standards.

## Supplementary material

10.2196/83774 Multimedia Appendix 1 Questionnaire content.

10.2196/83774 Multimedia Appendix 2 Breakdown of the survey participants—roles and professions.

## Abbreviations

ADaM: Analysis Data Model
ARO: academic research organization
CDASH: Clinical Data Acquisition Standards Harmonization
CDISC: Clinical Data Interchange Standards Consortium
CRO: contract research organization
PMDA: Pharmaceuticals and Medical Devices Agency
SDTM: Study Data Tabulation Model
