# Supplementary material for: Current Status, Challenges, and Opportunities Associated With Implementing Clinical Data Interchange Standards Consortium Standards in Japanese Academic Medical Centers: Cross-Sectional Survey
Source: JMIR Med Inform. 2026 Mar 6;14:e83774. doi: 10.2196/83774 (PMC12978922; doi:10.2196/83774)
Supplement: Multimedia Appendix 1 [file medinform-v14-e83774-s001.docx]

# Appendix 1. Questionnaire content

# A Questionnaire Survey Regarding the State of Dissemination of CDISC Standards

**Respondent's Email Address:**

**Respondent's Name (Text):**

**Respondent's Facility Name (Radio Button):**

**Respondent's Roles and Professions (Multiple Selections Allowed):**

- Data Management (DM)
- Biostatistics
- CDISC Specialist
- Medical Support (Physician)
- Medical Writing
- IT
- Regulatory Affairs
- Department Head
- Other

### **Experience with Contracted CDISC Standards Data Creation(Multiple Answers Allowed):**

Even if CDISC data creation was outsourced to a third party, it is included in the contract history.

- Experience in industry-sponsored clinical trials for drug approval
- Experience in investigator-initiated clinical trials for drug approval
- Experience in specified clinical trials under Clinical Research Act
- Experience in other clinical research

### **Implementation Status of CDISC Standards (Multiple Answers Allowed):**

Implementation refers to creating CDISC data using internal resources or, in the case of outsourcing, conducting acceptance work by CDISC experts.

- CDASH
- SDTM
- ADaM
- ODM

### **Barriers to CDISC Standard Implementation (Multiple Answers Allowed):**

- No need to implement CDISC standards
- Outsourced, hence no need
- Using standards other than CDISC
- Lack of personnel knowledgeable in CDISC
- High human and financial effort; lack of resources
- Unsure how to implement CDISC standards
- Want to learn CDISC but don't know how
- Limited Japanese materials
- Time-consuming translation of terms
- Other barriers

### **Future Policies Regarding CDISC Standards-Compliant Data Creation (Single Choice):**

- Will undertake, and the work will be performed internally
- Will undertake, and both internal and external personnel will perform the work
- Will undertake, and external experts will perform the work with internal involvement
- Will undertake, but only external experts will perform the work without internal involvement
- Will not undertake
- No decision made

### **Availability of CDISC Standards-Related Documents and Training (Multiple Answers Allowed):**

Documents and services owned by your institution

- Japanese-language educational materials on CDISC standards
- Documents on implementation know-how for CDISC standards
- Tools supporting CDISC standards implementation
- Beginner-level training on CDISC standards

### **Ability to Provide CDISC Standards-Related Documents and Training (Multiple Answers Allowed):**

Documents and services that can be provided when other institutions implement the CDISC standard

- Japanese-language educational materials on CDISC standards
- Documents on implementation know-how for CDISC standards
- Tools supporting CDISC standards implementation
- Beginner-level training on CDISC standards

### **Expectations for Pharmaceutical Companies, CROs, and Other AROs (Multiple Answers Allowed):**

- Desire for training sessions (seminars, workshops, etc.) on CDISC
- Desire for On-the-job training (OJT) related to CDISC
- Desire for materials and information on CDISC

### **Ability to Provide to Pharmaceutical Companies, CROs, and Other AROs (Multiple Answers Allowed):**

- Able to host training sessions (seminars, workshops, etc.) on CDISC
- Able to provide On-the-job training (OJT) related to CDISC
- Able to provide materials and information on CDISC

### **Number of Data Managers:**

*Count non-regular and part-time staff as one person each. For those with multiple roles, count each role separately.* *Enter "0" if there are no personnel in charge.*

Data Manager (DM) (Number of People): _____

### **Number of Biostatisticians:**

*Count non-regular and part-time staff as one person each. For those with multiple roles, count each role separately.* *Enter "0" if there are no personnel in charge.*

Biostatistician (Number of People): _____
