# Supplementary material for: Current Status, Challenges, and Opportunities Associated With Implementing Clinical Data Interchange Standards Consortium Standards in Japanese Academic Medical Centers: Cross-Sectional Survey
Source: JMIR Med Inform. 2026 Mar 6;14:e83774. doi: 10.2196/83774 (PMC12978922; doi:10.2196/83774)
Supplement: Multimedia Appendix 2 [file medinform-v14-e83774-s002.docx]

Appendix 2. Breakdown of the Survey Participants: Roles and Professions

| Occupation | Number of Responses  (n=84) | Percentage  (%) |
| --- | --- | --- |
| Data Manager（DM） | 29 | 34.5% |
| Other | 16 | 19.0% |
| Department Head | 8 | 9.5% |
| Biostatistician | 6 | 7.1% |
| Medical Support (Physician) | 2 | 2.4% |
| Data Manager（DM）, Biostatistician, CDISC Specialist | 2 | 2.4% |
| Data Manager（DM）, Biostatistician | 2 | 2.4% |
| Data Manager（DM）, IT | 2 | 2.4% |
| Regulatory Affairs, Department Head | 1 | 1.2% |
| Department Head, Other | 1 | 1.2% |
| Biostatistician, Department Head | 1 | 1.2% |
| Biostatistician, IT, Department Head | 1 | 1.2% |
| Medical Support (Physician), Department Head | 1 | 1.2% |
| Medical Writing, Department Head | 1 | 1.2% |
| Data Manager（DM）, Department Head | 1 | 1.2% |
| Data Manager（DM）, Biostatistician, IT, Department Head | 1 | 1.2% |
| Data Manager（DM）, Biostatistician, IT | 1 | 1.2% |
| Data Manager（DM）, Medical Support (Physician) | 1 | 1.2% |
| Data Manager（DM）, Other | 1 | 1.2% |
| Data Manager（DM）, IT, Department Head | 1 | 1.2% |
| Data Manager（DM）, CDISC Specialist, Department Head | 1 | 1.2% |
| Data Manager（DM）, CDISC Specialist | 1 | 1.2% |
| IT, Department Head | 1 | 1.2% |
| IT, Other | 1 | 1.2% |
| IT | 1 | 1.2% |

Note: Breakdown of the "Other" category (n=16) The respondents classified as "Other" consist of the following job functions:

Administrative Staff / Secretariat: 8 (e.g., Clinical trial secretariat, General affairs, IRB secretariat)

Center Directors / Faculty Members: 4 (e.g., Professor, Associate Professor, Lecturer, Vice-center director)

Specialized Professionals: 4 (e.g., Clinical Research Coordinator [CRC], Project Manager [PM], Quality Manager [QM])
